# Brd4 Activates Early Viral Transcription upon Human Papillomavirus 18 Infection of Primary Keratinocytes

**DOI:** 10.1128/mBio.01644-16

**Published:** 2016-11-22

**Authors:** Caleb C. McKinney, Min Jung Kim, Dan Chen, Alison A. McBride

**Affiliations:** Laboratory of Viral Diseases, National Institute of Allergy and Infectious Diseases, National Institutes of Health, Bethesda, Maryland, USA

## Abstract

Human papillomaviruses (HPVs) replicate in the cutaneous and mucosal epithelia, and the infectious cycle is synchronous with the differentiation program of the host keratinocytes. The virus initially infects dividing cells in the lower layers of the epithelium, where it establishes a persistent infection. The viral genome is maintained as a low-copy-number, extrachromosomal element in these proliferating cells but switches to the late stage of the life cycle in differentiated cells. The cellular chromatin adaptor protein Brd4 is involved in several stages and processes of the viral life cycle. In concert with the viral transcriptional regulator E2, Brd4 can repress transcription from the early viral promoter. Brd4 and E2 form a complex with the viral genome that associates with host chromosomes to partition the viral genome in dividing cells; Brd4 also localizes to active sites of productive HPV DNA replication. However, because of the difficulties in producing HPV viral particles, the role of Brd4 in modulating viral transcription and replication at the initial stage of infection is unclear. In this study, we have used an HPV18 quasivirus-based genome delivery system to assess the role of Brd4 in the initial infectivity of primary human keratinocytes. We show that, upon infection of primary human keratinocytes with HPV18 quasivirus, Brd4 activates viral transcription and replication. Furthermore, this activation is independent of the functional interaction between Brd4 and the HPV18 E2 protein.

## IMPORTANCE

HPVs lack encapsidated proteins and so rely exquisitely on host cellular factors to initiate their gene expression programs in newly infected cells. Brd4 is an important cellular chromatin adaptor molecule that normally activates host transcription initiation and elongation. In this study, we further optimize and utilize a quasivirus infection system to show that Brd4 activates HPV18 transcription at early stages of infection. HPVs are important human pathogens causing a wide range of cutaneous and tumorigenic morbidities. Therefore, specifically targeting this protein could provide a new target of therapeutic prevention of establishment of HPV infections.

## INTRODUCTION

Human papillomaviruses (HPVs) are an ancient group of nonenveloped, double-stranded, circular DNA viruses that exhibit exquisite species specificity and mucosal or cutaneous epithelial cell tropism. A subset of mucosotropic human papillomaviruses are categorized as high risk oncogenic (such as HPV16, -18, and -31) and are leading etiological agents of nearly all cervical cancers and a growing number of anogenital and oropharyngeal carcinomas. The viral life cycle is highly attuned to human epithelial cell differentiation. It is generally accepted that incoming viral particles infect the basal layer of a stratified epithelium through a microabrasion or through exposed squamocolumnar junction cells in the transformation zone of the cervix (1). Infected cells must undergo mitosis and cell division for the virus to access the nucleus (2, 3), and cellular proliferation that occurs during the wound healing process would facilitate entry of viral DNA into the host nucleus. Within the nucleus, initiation of a viral gene expression program produces the E1 and E2 replication proteins, which are required to replicate the incoming viral genome to low levels to establish infection (4) In the next stage of infection, the viral genome replicates in synchrony with the host DNA and is partitioned to daughter cells. When these infected cells move upward through the epithelium and differentiate, late transcription is activated, leading to a massive upregulation of the E1 and E2 viral replication proteins, with a resulting amplification of viral genomes to a high copy number. Finally, capsid proteins are synthesized, and virions assembled, in the most superficial layers of the epithelium (5).

Although the HPV life cycle can be recapitulated in organotypic three-dimensional (3D) raft cultures to produce native virions (6), it is difficult to obtain high yields, and mutant genomes cannot be packaged unless they can complete the infectious cycle. Studies of infectivity often employ pseudovirions composed of recombinantly produced HPV L1 and L2 capsid proteins containing a reporter plasmid (7). Pseudovirus production overcomes the challenges of mimicking the viral life cycle in cell culture and provides an amenable *in vitro* system that has been used extensively to study capsid morphology, intracellular trafficking, and neutralization (8–10). However, L1/L2 capsids containing recombinant HPV genomes, quasiviruses, can also be produced to high titers (11). In this study, we have optimized HPV18 quasivirus production and infection of primary keratinocytes and have used the system to identify the role of the Brd4 chromatin adaptor protein in viral transcription and replication at very early stages of infection.

The viral E2 protein is multifunctional and essential for several stages of the infectious cycle; it is required for viral transcription (both activation and repression), initiation of viral DNA replication in concert with the viral E1 protein, and persistent replication and partitioning of extrachromosomal viral genomes (reviewed in reference 12). The cellular protein Brd4 is one of the major interactors with the papillomavirus E2 proteins (13–16). Brd4 is a member of the BET (bromodomain and extraterminal domain) family of chromatin binding proteins that bind the acetylated tails of H3 and H4 histones (17) and recruit transcription initiation factors and facilitate transcriptional elongation (18). Brd4 is involved in viral transcription and replication, although the precise roles of Brd4 in the HPV life cycle are complex and somewhat controversial (reviewed in reference 19).

The oncogenic alphapapillomavirus (alpha-PV) E2 proteins can activate viral transcription through consensus sites that are located upstream from promoter elements (20), but binding to sites that are proximal to the early viral promoter represses transcription (21). Both E2-mediated activation and repression require interaction with the Brd4 protein (13, 22–25). The C-terminal domain of Brd4 interacts with two highly conserved residues of E2 (R37 and I73 in most E2s and R41 and I77 in HPV18) (14, 26) that are required for the transcriptional activation and repression functions of E2 but are not required for the role of E2 in initiation of replication. Brd4 stimulates elongation of RNA polymerase II transcription by recruiting pTEFb to promoter regions (18). pTEFb also binds to the C-terminal region of Brd4, and this interaction can be antagonized by E2 (27). Most studies of Brd4-dependent repression have analyzed the effect of E2 on transcription from integrated HPV genomes in cervical carcinoma-derived cell lines. Less is known about the transcriptional effect of E2 on replicating extrachromosomal genomes, and one study indicates that these genomes may be more impervious to the repressive function of E2 (28).

E2 proteins that are unable to bind Brd4 (mutated in R37/I73 residues) support transient viral replication, indicating that Brd4 is not required for this process (15, 29–31). Nevertheless, Brd4 is recruited to, and is essential for the formation of, nuclear replication foci that are formed by the E1 and E2 proteins (32–34). This localization likely reflects the interaction of the replication foci with host chromatin, rather than a direct role for Brd4 in viral DNA synthesis (33, 35). Furthermore, it is likely that these replication foci represent the late phase of the viral life cycle, when E1 and E2 are expressed to productively amplify viral DNA.

In addition to its transcription and replication initiation functions, E2 binds and tethers viral genomes to host mitotic chromosomes to partition them to daughter cells (36–39). Brd4 colocalizes with the E2 proteins from many PVs in prominent speckles on mitotic chromosomes (14, 15, 26, 40). These targets are regions of the host chromosomes that are susceptible to replication stress and may link viral genome partitioning to viral DNA replication (35).

Despite evidence that the interaction of E2 and Brd4 is highly conserved and can be observed in multiple processes and phases of the viral life cycle, there is also evidence that it is not essential (41). Stubenrauch and colleagues have shown that, when transfected into primary keratinocytes, HPV31 genomes encoding a Brd4-binding-defective E2 protein (I73L mutation) not only can replicate and be maintained extrachromosomally but also are amplified and express late transcripts in differentiated keratinocytes (22, 41).

In this study, we optimized HPV18 quasivirus production and infection of primary keratinocytes. Thereafter, we used this system to study the role of Brd4 upon initial infection, a relatively understudied phase of the infectious cycle. The quasivirus system allowed us to study early transcription and replication of a viral genome, delivered to the nucleus at a low level and by natural processes. Moreover, the system allows us to package mutant genomes to elucidate the role of viral protein functions early in infection. We demonstrate that Brd4 activates early viral transcription upon infection and does so in an E2-independent manner.

## RESULTS

### Production of HPV18 quasiviruses.

Previous studies have shown that HPV16 and HPV31 genomes can be packaged in recombinant HPV16 virus-like particles (VLPs), and the resulting quasiviruses can infect keratinocyte cell lines, such as HaCaT (11). We have also shown that HPV18 genomes can be packaged in HPV18 VLPs, and these HPV18 quasiviruses can infect primary keratinocytes (42). However, because HPV16 is the best-characterized pseudovirus for infectivity and neutralization studies (43), we chose to package HPV18 genomes in an HPV16 VLP to generate an HPV16-18 quasivirus. HPV18 genomes were chosen because they replicate very efficiently and proficiently immortalize keratinocytes (44).

**FIG 1:**
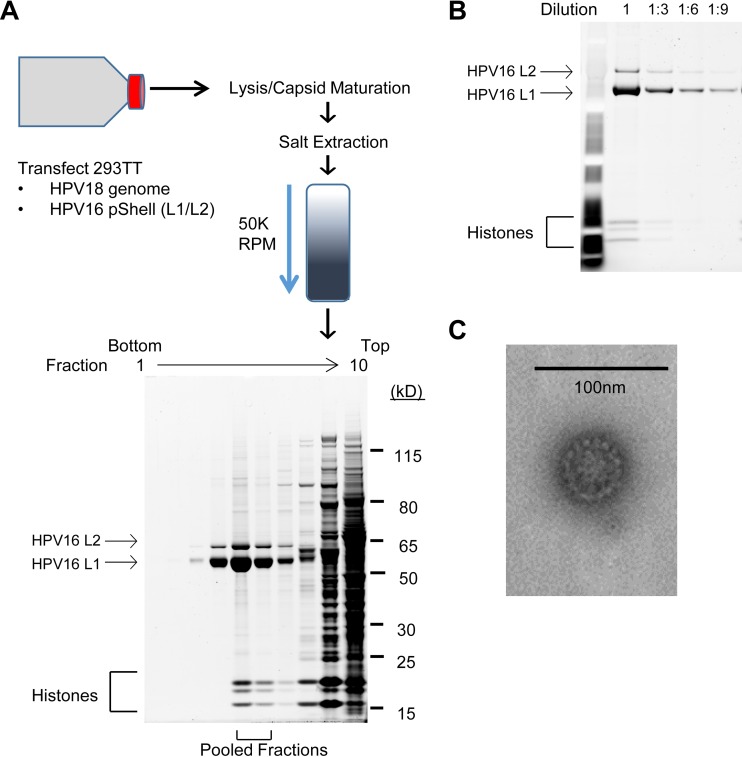
Overview of quasivirus stock production and analysis. (A) 293TT cells were cotransfected with recircularized HPV18 genome and an HPV16 L1/L2 capsid protein expression plasmid. Forty-eight hours posttransfection, cells were lysed and virions were extracted in high-salt buffer. Clarified lysates were separated through a 27 to 39% OptiPrep density gradient by high-speed centrifugation. Fractions were collected, and total protein in each was resolved by SDS-PAGE and visualized by SYPRO Ruby staining. Fractions that contained predominantly L1 and L2 and cellular histones were pooled as a virus stock. (B) Pooled virus stocks were diluted and resolved by SDS-PAGE to qualitatively and quantitatively determine capsid amount and L1/L2 ratio. (C) Electron micrograph of 50-nm quasivirus particles.

HPV16-18 quasiviruses were generated by transfecting a plasmid encoding the HPV16 L1 and L2 protein, along with recircularized HPV18 genomes, into 293TT cells. To evaluate the quasiviruses, virus preparations isolated by OptiPrep density gradient purification were subjected to SDS-PAGE and L1/L2 protein quantities were calculated with reference to a standard curve of decreasing concentrations of bovine serum albumin (BSA) (Fig. 1A and B). Quantitative and qualitative analysis of L1 and L2 protein abundance revealed an L1/L2 ratio of about 9:12 (Fig. 1B), similar to that reported previously for pseudovirus (45). Electron microscopy (Fig. 1C) showed that pooled fractions contained viral particles that resemble papilloma virions (45). Quantitation of viral genome equivalents (VGE) demonstrated that 0.1% of VLPs contained a viral genome.

### HPV18 quasivirus infection of primary human foreskin keratinocytes.

Previous studies have shown that the HaCaT skin keratinocyte cell line is susceptible to infection with HPV31 raft-derived virus, as well as HPV16-HPV31 quasivirus (11). There have also been many studies analyzing infection by HPV pseudoviruses, which deliver a reporter plasmid to cells. However, we wanted to analyze host cellular processes that affect the early transcription and replication events that occur when the HPV genome is delivered to the nucleus by infection, and primary human keratinocytes are more fitting for these studies.

**FIG 2:**
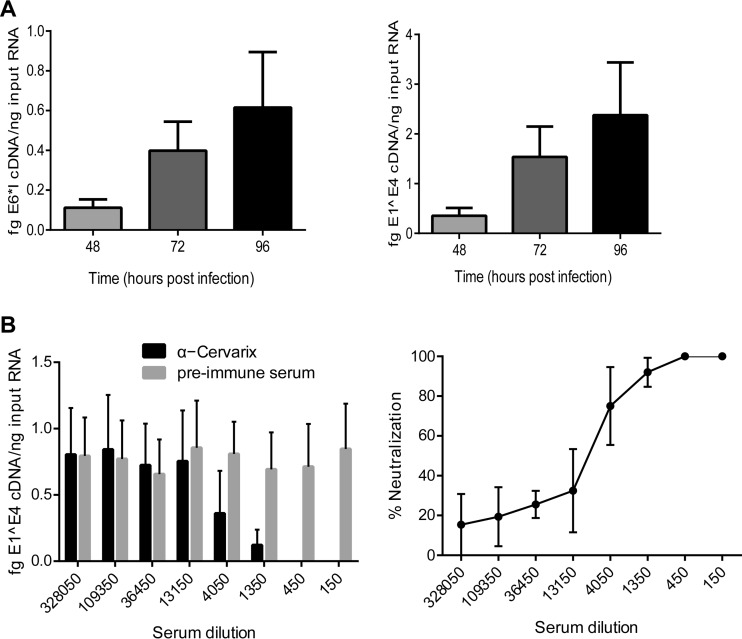
Infection of primary keratinocytes with HPV18 quasivirus and neutralization of infection with Cervarix antiserum. (A) Primary HFKs were infected with HPV18 quasivirus at 100 VGE/cell and incubated for the indicated times. E1^E4 and E6*I spliced early transcripts were detected by qRT-PCR and normalized to human TATA binding protein (TBP) transcripts. (B) Quasivirus was preincubated with the indicated dilutions of rabbit serum obtained pre- and post-vaccination with Cervarix. Primary keratinocytes were infected at 100 VGE/cell, and at 72 h postinfection, viral E1^E4 transcript abundance was determined with qRT-PCR and normalized to human TATA binding protein. Percent neutralization compared to preimmune serum is shown on the right. *n* = 3; error bars show standard errors of the means.

To this end, we infected early-passage human foreskin keratinocytes (HFKs) with HPV16 capsids containing HPV18 genomes and measured viral transcription (E1^E4 and E6*I transcripts) at 48, 72, and 96 h postinfection (p.i.). To ensure that we were measuring *de novo* viral transcription, primers were designed that were specific for spliced viral transcripts and would not detect contaminating DNA. As can be seen in Fig. 2A, both viral transcripts could be detected at 48 h postinfection and steadily increased thereafter.

### HPV18 quasivirus infection can be neutralized with Cervarix antiserum.

To prove that viral genomes (and subsequent transcription) resulted from capsid-mediated delivery, we neutralized infection with rabbit antisera generated against the commercially available HPV16 and HPV18 VLP subunit vaccine Cervarix. Quasivirus preparations were preincubated with serial dilutions of either preimmune serum or immune serum. In infections at 100 VGE/cell, E1^E4 spliced transcripts were reproducibly detected, and at Cervarix dilutions lower than 1:1,350, there was >90% reduction in HPV18 infectivity compared to preimmune serum (Fig. 2B). A relatively large amount of antibody was needed to neutralize HPV18 infectivity; however, HPV16 L1 and L2 typically assemble promiscuously around cellular DNA as well as cotransfected genomes (9); as calculated above, only 0.1% of HPV16 capsids contain viral genomes, and so the quasivirus preparation contains an excess of exposed neutralization epitopes.

In summary, we can detect HPV18 spliced transcripts upon infection of keratinocytes, and this infection can be neutralized by Cervarix specific antiserum. This confirms that delivery of the HPV18 genome, and initiation of the viral gene expression program, was mediated by HPV16 particles.

### HPV18 genomes do not replicate in 293TT cells, allowing the use of an HPV quasivirus infection early replication assay.

Our primary goal is to analyze the cellular processes that affect early HPV transcription and replication in primary keratinocytes. This requires highly sensitive and specific assays for newly replicated viral DNA and *de novo* viral transcripts. For viral transcripts, sensitivity can be achieved using quantitative PCR (qPCR), and specificity can be ensured using primers for spliced viral transcripts (see above). Viral DNA can also be measured by qPCR, but it is necessary to distinguish between input and newly replicated DNA. Traditionally, HPV replication has been measured by transfecting bacterially generated viral genomes (cleaved from the prokaryotic vector) into cells, and after a few days, isolating and digesting viral DNA with DpnI to remove residual bacterially methylated input DNA. However, we first had to determine whether HPV18 genomes could replicate in the packaging cell line 293TT before deciding whether the DpnI replication assay could be used to analyze replication of HPV18 genomes delivered by quasivirus.

**FIG 3:**
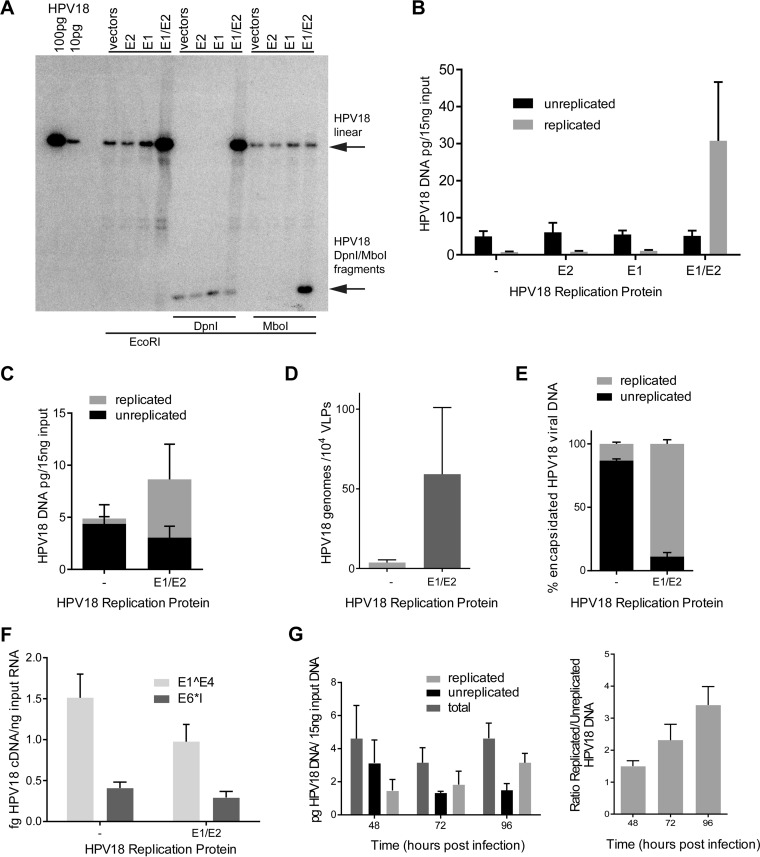
HPV18 replicates in 293TT cells only when E1 and E2 are coexpressed, enabling a DpnI resistance replication assay in quasivirus-infected keratinocytes. (A and B) Recircularized HPV18 genomes were cotransfected into 293TT cells along with pMEP E1 and E2 expression plasmids. Replicated DNA is resistant to DpnI and sensitive to MboI cleavage. 293TT cells replicate the HPV18 genome only in the presence of exogenously expressed E1 and E2 proteins, as shown by Southern blot analysis (*n* = 2) (A) and qPCR (*n* = 3) (B). (C) E1 and E2 expression enhances HPV18 replication in cells cotransfected with the pSHELL plasmid and actively packaging viral genome (*n* = 3). (D) HPV18 genome-containing capsids are enriched in replicated viral DNA when produced from E1/E2-expressing cells (*n* = 3). (E) The packaging efficiency of HPV18 genomes is greatly increased when produced from E1/E2-expressing cells (*n* = 3). (F) Quasiviruses containing replicated and unreplicated genomes have similar transcriptional activities upon HFK infection (*n* = 3). (G) Quasiviruses containing unreplicated viral genomes allow analysis of nascently produced viral DNA after infection of primary HFKs. Total and replicated (DpnI-resistant) DNA was detected by qPCR and normalized to β-actin (*n* = 3). (A to E) Forty-eight hours posttransfection. (F and G) Seventy-two hours postinfection. Error bars show standard errors of the means. A paired *t* test was used for statistical analysis.

To this end, 293TT cells were transfected with recircularized HPV18 genomes, and 48 h later, total DNA was isolated and analyzed for HPV replication using the DpnI resistance assay and Southern blot analysis. This showed that HPV18 genomes cotransfected with empty pMEP vectors, or pMEP vectors expressing just one of the replication proteins (E1 or E2), were unable to replicate in 293TT cells (Fig. 3A and B). However, when expression vectors for both E1 and E2 were cotransfected with the HPV18 genomes, robust replication could be observed. To further prove that HPV18 could replicate only in the presence of E1 and E2 expression plasmids, DNA was digested with MboI, which will digest bacterially produced DNA only if it has replicated in eukaryotic cells and has lost the methylation marks. As before, HPV18 replicated in 293TT cells only in the presence of exogenously expressed E1 and E2 proteins. Thus, the DpnI resistance assay can be used to determine whether quasivirus containing HPV18 packaged in 293TT cells can replicate in the target cells.

These data also indicate that 293TT cells are able to support replication of HPV genomes but that they are unable to express the replication proteins E1 and E2. We noted that exogenous expression of E1 and E2 resulted in robust replication of HPV18 and hypothesized that this might increase packaging efficiency (though the resulting quasivirus could not be used to study early viral replication using the DpnI resistance assay, because the genomes would now be susceptible to DpnI digestion). The pMEP vectors used to express E1 and E2 are >10 kb in size and will not be packaged because they are above the 8-kb packaging limit (8, 46). Furthermore, despite the fact that E1 and E2 are expressed from the inducible metallothionein promoter, leaky expression ensures that they are expressed even in the absence of heavy metal induction (47).

To package replicated HPV18 DNA, the genome was cotransfected into 293TT cells along with E1/E2 and L1/L2 expression constructs. As seen before, transfected 293TT cells contain DpnI-sensitive viral DNA; however, E1 and E2 coexpression results in the robust appearance of DpnI-resistant DNA (Fig. 3C). Furthermore, as shown in Fig. 3D, quasivirus stocks purified from HPV replication-competent packaging cells showed a 20-fold increase in packaging efficiency. To verify that this enrichment was due to replicated HPV18 genomes, encapsidated DNA was purified from HPV16 capsids, digested with DpnI, and analyzed by qPCR. Indeed, virus preparations from replication-competent cells contain approximately 90% replicated HPV DNA (Fig. 3E), even though this DNA comprised only about 60% of the DNA present in 293TT cells. Thus, replicated genomes are selectively enriched in HPV16 capsids. To determine whether unreplicated or E1/E2 replicated HPV18 genomes delivered by quasivirus had different infectivities, HFKs were infected with 100 VGE/cell from each virus preparation. Seventy-two hours after infection, cells were harvested and assayed for expression of early viral transcripts. As shown in Fig. 3F, there was no significant difference in infectivity (as measured by viral transcription) between viruses that contained replicated genomes and those that contained nonreplicated genomes. Thus, enhancing encapsidation or packaging replicated genomes does not substantially alter infectivity when cells are infected with equivalent VGEs.

In summary, we can package replicated HPV18 genomes, resulting in a much greater packaging efficiency and effective viral titer. Alternatively, we can generate quasivirus containing unreplicated HPV18 viral genomes that can be used in the DpnI resistance assay for studies of early events of replication upon infection of keratinocytes. To this end, we infected early-passage HFKs with HPV16 capsids containing unreplicated HPV18 genomes and measured *de novo* viral DNA replication by qPCR at 48, 72, and 96 h postinfection. As had been seen for viral transcription in Fig. 2, replicated viral DNA could be detected as early as 48 h and steadily increased through 96 h (Fig. 3G).

### Brd4 depletion decreases HPV18 early gene transcription.

**FIG 4:**
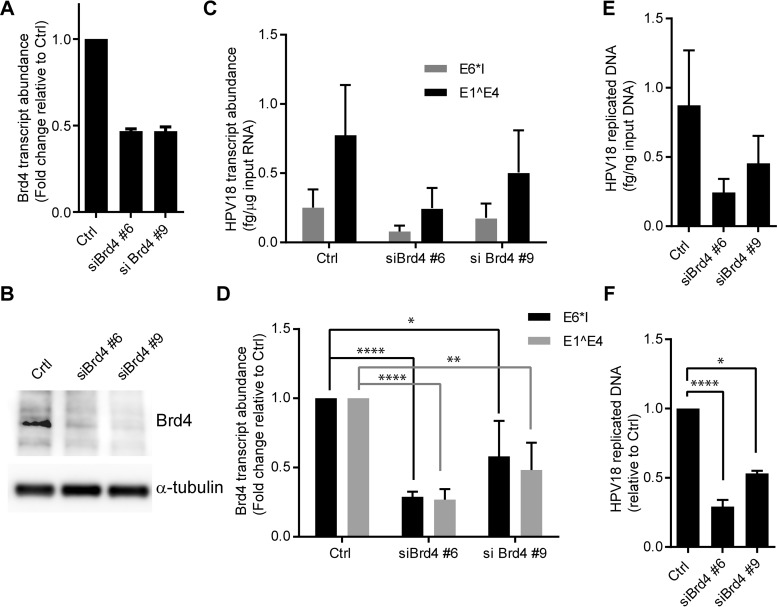
Brd4 depletion reduces HPV18 transcript abundance and viral DNA replication in infected HFKs. (A and B) HFKs were transfected with 20 nM Brd4-targeting or All* negative-control siRNA. Twenty-four hours posttransfection, cells were infected with 100 VGE/cell of HPV18 quasivirus. Seventy-two hours postinfection, cells were harvested for RNA, DNA, and protein. Knockdown efficiency was determined by Brd4 RNA (A) and Brd4 protein abundance (B). Brd4 protein levels were determined by immunoblotting, and α-tubulin is provided as a control for equal loading. Cell growth was monitored in an IncuCyte microscope to assess toxicity of the Brd4 downregulation (see Fig. S1 and Movie S1 in the supplemental material). (C and D) HPV18 E1^E4 and E6*1 transcripts were detected by qRT-PCR, corrected to TATA binding protein (TBP) transcripts (C), and normalized to siCtrl (D). *n* = 5. (E and F) Total DNA was digested with DpnI, and the abundance of nascently replicated HPV18 genomes was detected by qPCR, corrected to β-actin (E), and normalized to siCtrl (F). *n* = 3. Error bars show standard errors of the means. A paired *t* test was used for statistical analysis (*, *P* < 0.05; **, *P* < 0.01; ***, *P* < 0.001; ****, *P* < 0.0001).

The Brd4 protein has been shown to be involved in viral transcription and replication at different phases of the life cycle; however, its role at the onset of viral gene expression immediately after infection has not been studied. Therefore, to determine the role of Brd4 in HPV18 gene expression at very early stages of infection, subconfluent HFKs were depleted of Brd4 with two different small interfering RNAs (siRNAs) and infected 24 to 48 h later with 100 VGE/cell of HPV18 quasivirus. At 72 h postinfection, total cellular RNA was isolated and analyzed for HPV18 E1^E4 and E6*I spliced early transcripts and Brd4 mRNA. siRNA treatment resulted in 50% depletion of Brd4 at the mRNA level (Fig. 4A). To ensure that Brd4 protein abundance was reduced by siRNA treatment, total cellular protein lysates were resolved by SDS-PAGE and immunoblotted with Brd4-specific antisera (Fig. 4B). To ensure that depletion of Brd4 was not detrimental to cell growth, cells were monitored by live cell microscopy for the 72-h period of infection. Figure S1 and Movie S1 in the supplemental material show the growth of cells undergoing Brd4 depletion.

When viral RNA was measured under these conditions, Brd4 depletion reduced the abundance of both early viral transcripts by 50 to 75% (Fig. 4C and D). Thus, Brd4 is an activator of HPV18 gene expression at early times after infection.

### Brd4 depletion reduces HPV18 replication.

To determine if Brd4 also affects early viral DNA replication, Brd4-depleted primary keratinocytes were infected as described above. These replication assays used virions containing unreplicated HPV18 genomes in order to monitor HPV genome abundance changes that stem from *de novo* DNA replication. Total cellular genomic DNA was prepared after 72 h of infection. HPV18 DNA abundance was quantified by the DpnI resistance qPCR assay. Indeed, Brd4 depletion reduced DpnI-resistant HPV18 genome abundance by about 50%, similar to the reduction seen for early viral transcripts (Fig. 4E and F). To ensure that the depletion of Brd4 did not impact quasiviral infection and entry, we also measured total viral DNA and unreplicated viral DNA at the 72-h point. As shown in Fig. S2 in the supplemental material, there was no significant difference in the amount of unreplicated viral DNA after Brd4 depletion. Therefore, Brd4 promotes efficient *de novo* HPV18 replication in newly infected primary human keratinocytes.

### Brd4 enhancement of early HPV18 transcription is not E2 dependent.

**FIG 5:**
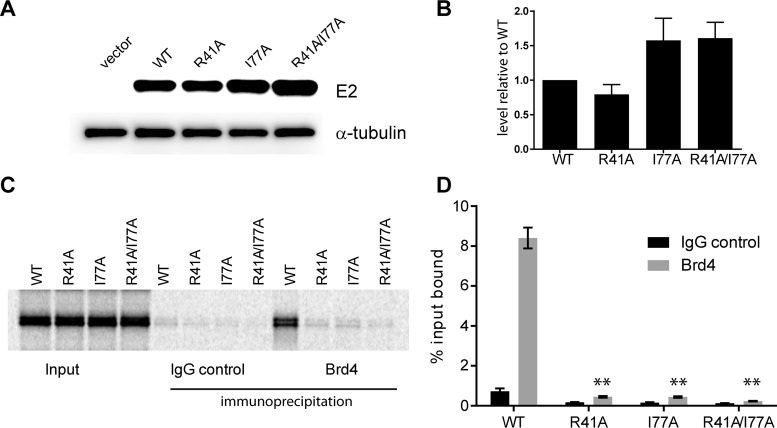
Expression and Brd4 binding of E2 mutants. (A) Western blot analysis of protein extracts from C33A cell lines stably transfected with pMEP4 (no E2), wild-type pMEP-E2 (WT), or mutated pMEP-E2 (R41A, I77A, and R41A/I77A). E2 proteins were tagged with a FLAG epitope and detected with FLAG-M2 monoclonal antisera. α-Tubulin is provided as a control for equal cell number. (B) E2 expression was corrected to tubulin expression and normalized to WT. Raw band volume was quantified by Gene Tools software (Syngene). *n* = 3. Error bars represent standard errors of the means. (C) ^35^S-labeled *in vitro*-translated E2 proteins were mixed with *in vitro*-translated Brd4 and immunoprecipitated with the Brd4-specific antibody 2290. Immune complexes were eluted and resolved by SDS-PAGE. Shown is a representative audioradiograph. (D) E2 bands were detected by using a Typhoon phosphorimager and quantified with Gene Tools software (Syngene). Brd4-bound E2 was corrected for background binding to rabbit IgG and normalized to WT. *n* = 3. A paired *t* test was used for statistical analysis (*, *P* < 0.05; **, *P* < 0.01; ***, *P* < 0.001; ****, *P* < 0.0001).

Brd4 is important for viral transcription, and this is thought to be mediated primarily through interaction with the E2 protein. This interaction has been well defined, and the contact between the C-terminal tail of Brd4 and the transactivation domain of E2 has been characterized both structurally and functionally (reviewed in reference 12). Two residues on one face of the transactivation domain of E2, R37 and I73, make direct contact with the Brd4 protein (26). Replacement of these residues with alanines (R37A and I73A) abrogates the transcriptional activation or repressive function of E2 but leaves replication intact (these residues are on the opposite face of the transactivation domain from the E1-interacting region). The R37 and I73 residues are highly conserved among all papillomaviruses, and replacement of these residues severely impairs the association of E2 with Brd4 in all E2 proteins tested (reviewed in reference 12). Therefore, equivalent alanine substitutions were generated in HPV18 E2 (R41A and I77A). To ensure that the mutated proteins were stable *in vivo*, C-33A cells expressing FLAG-tagged versions of the E2 proteins were established (Fig. 5A and B). Western blot analysis showed that each protein was expressed at least as well as the wild-type E2 protein, with levels of the I77A and double-mutated E2 proteins being slightly elevated compared to the wild type (WT). To ensure that the mutations abrogated the E2-Brd4 interaction, the mutated E2 proteins were tested for Brd4 binding (Fig. 5C and D). As shown, alanine substitution of either R41 or I77 was sufficient to abrogate almost all binding of E2 to Brd4. Any residual binding was completely eliminated by the double amino acid substitution.

**FIG 6:**
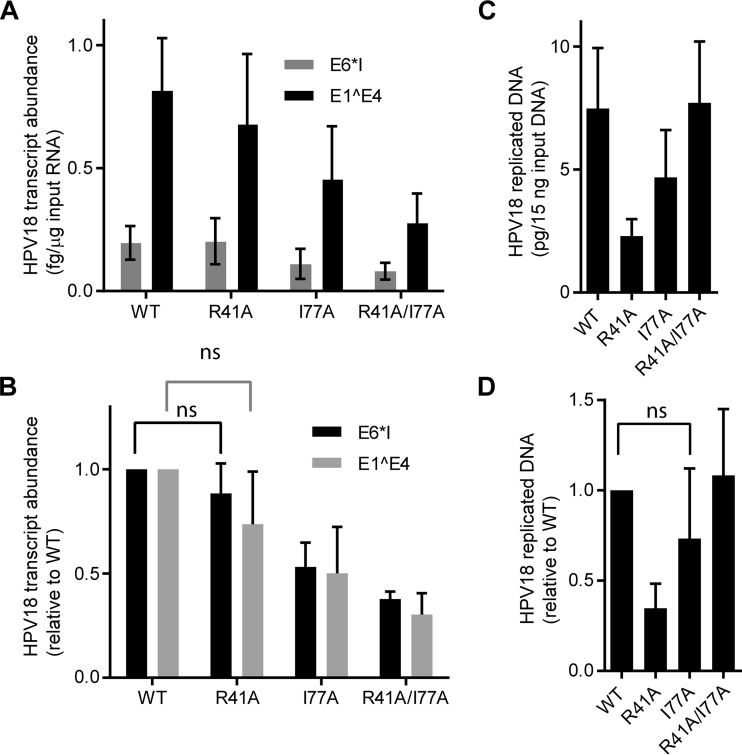
Efficient gene expression is not repressed by the Brd4 binding function of HPV18 E2 upon *de novo* infection. Quasivirus inocula containing WT HPV18 genomes or genomes mutated in one or both of the key Brd4 binding E2 transactivation domain residues were used to infect HFKs at 100 VGE/cell. (A and B) E1^E4 and E6*1 early spliced transcripts were measured by qRT-PCR 72 h postinfection, corrected to TATA binding protein (TBP) transcripts (A), and normalized to WT (B). *n* = 3. Error bars show standard errors of the means. (C and D) Total DNA was digested with DpnI, and the abundance of newly replicated HPV18 genomes was detected by qPCR and normalized to β-actin. *n* = 3. Error bars show standard errors of the means. A paired *t* test was used for statistical analysis; ns, not significant.

The mutated E2 codons were then incorporated into the background of the complete viral genome, and the genomes were packaged into HPV16 quasivirus particles. Subconfluent primary HFKs were infected at 100 VGE/cell, and total RNA and DNA were harvested at 72 h postinfection (p.i.). As shown in Fig. 6, viral transcription was largely unaffected by the R41A mutant genome, indicating that the E2-Brd4 interaction was not required for transcription at this stage of infection. Transcription was reduced by 40 to 50% from the I77A genome and even more so from the double mutant, but we have to assume that this is due to other effects on E2 structure or interactions as R41A is equally defective in Brd4 binding. Furthermore, the R41A E2 protein is expressed at levels most similar to the WT (Fig. 6A and B). R41A was consistently reduced in its ability to support early DNA replication, but this cannot be solely due to the inability to interact with Brd4 as I77A and R41/I77A genomes replicate at levels close to the wild type but are defective for Brd4 binding. We conclude that immediate early HPV18 transcription and replication are not dependent on the E2-Brd4 interaction.

## DISCUSSION

In this study, we generated HPV18 quasivirus to study the early steps of viral transcription and replication upon infection of primary keratinocytes. Quasivirus preparations are relatively easy to produce and have advantages over “native” virus produced in organotypic rafts. For example, mutant viral genomes can be packaged as efficiently as wild-type genomes. Additionally, as we show here, HPV genomes do not replicate in the producer 293 cell line (unless E1 and E2 are coexpressed), and so nascent DNA replication can be detected and quantified using a DpnI resistance assay. Although viral genomes do not replicate in 293 cells, they are packaged in host chromatin and so are delivered to keratinocytes in a way that mimics authentic viral particles. We show that viral transcription and replication can be detected at 48 h postinfection and increase steadily up to 96 h (the latest time point measured). Furthermore, infection can be abrogated by neutralizing antiserum.

The quasivirus infection system was used to determine the role of the Brd4 protein in the earliest steps of HPV infection. The role of Brd4 in HPV infection has been well studied, though with some conflicting results about the importance of the interaction of E2 with Brd4 at different stages of the life cycle. Using siRNA to downregulate Brd4 expression, we show that Brd4 activates HPV18 transcription at early stages of infection. This is perhaps not surprising since Brd4 is an activator of host transcriptional initiation and elongation, but most studies have shown that, in the presence of the viral E2 protein, Brd4 represses viral transcription from the viral early promoter. However, HPV virions do not contain any viral proteins other than the major and minor capsid proteins, L1 and L2. Therefore, immediate early transcription is completely dependent on cellular transcription factors, and Brd4 serves to activate this transcription.

To determine whether the E2 protein modulated early Brd4-dependent viral transcription, we packaged mutant genomes that contained amino acid substitutions in two highly conserved E2 residues that have been shown to be involved in E2-Brd4 complex formation. These mutations, R41A and I77A, each individually abrogated the E2-Brd4 interaction. However, there was no significant effect of the R41A E2 substitution on early viral transcription, even although the E2 protein could not bind Brd4. We conclude that transcriptional activation of early viral transcripts by Brd4 is independent of its interaction with E2. E2 proteins mutated in residues analogous to R41A or I77A, and unable to bind Brd4, are also able to very efficiently support initiation of HPV replication in transient-transfection assays. However, Brd4 may play more indirect roles in replication by anchoring viral genomes or replication foci to host chromatin (32, 34, 48). Here, we show that HPV18 viruses encoding E2 proteins unable to bind Brd4 can replicate efficiently, indicating that interaction with chromatin is not essential at this early stage of infection.

During the initial HPV infection, the E1 and E2 viral replication proteins are required at low levels to initiate a low-level replication program and establish extrachromosomal genomes in proliferating cells. Ozbun demonstrated that a transcript encoding E2 is one of the first detected after infection with native HPV31 virions (4), and consistent with this, we can detect viral replication at 48 h postinfection (the earliest time point). It is also thought that at low levels E2 can activate viral transcription through the distal E2BS4 while at higher levels it can repress through binding sites proximal to the promoter (20). The very early HPV31 E2 transcript may be transcribed from an alternative promoter in the early coding region, which would allow E2 synthesis to support early replication from a promoter that is impervious to E2 repression. We have been unable to detect a role for E2BS4 in early HPV18 quasivirus infection (data not shown). Collectively, these findings suggest that E2 primarily supports viral genome replication, as opposed to regulating viral transcription, at early times after infection.

Notably, Brd4 depletion directly repressed HPV18 transcription and reduced DNA replication in quasivirus-infected cells. Restricting transcription of the viral replication proteins may have a rate-limiting impact on viral DNA replication processes at the onset of infection, or Brd4 might promote viral DNA replication in an E2-dependent manner. Brd4 is recruited to HPV replication foci formed by the E1 and E2 proteins (32, 34, 48), but its role is somewhat unclear. Some studies find that Brd4 is required for replication and focus formation, but others show that in the presence of actively replicating DNA, Brd4 is displaced and not required for replication (32, 34, 48). HPV31 genomes containing a very conservative substitution in E2 (I73L) that abrogates Brd4 binding can be maintained as episomes (41, 48), amplify in differentiated cells, and express late transcripts (albeit at a reduced level) (41). Therefore, Brd4 does not appear to be absolutely essential for viral replication but likely enhances the process, perhaps by localizing viral genomes to beneficial regions of the host nucleus (35, 49, 50). However, this could also vary among viral types: for example, Gauson and colleagues confirm that an HPV31 genome with an E2 I73A mutation is functional but an HPV16 genome with the same mutation is unable to persist as an episome (34). The strongest evidence that the E2-Brd4 interaction is important for some stages of the infectious HPV cycle is the fact that the E2 residues important for Brd4 interaction (residues 41 and 77 in HPV18) are highly conserved in virtually all papillomaviruses (12).

In summary, we have shown that Brd4 activates immediate early transcription and initial replication of genomes delivered to the nucleus of primary keratinocytes in a virus particle. This activation is independent of the viral E2 protein, which seems primarily involved in amplification of viral DNA at this time period. It will be important to understand if targeting Brd4 directly can cripple the establishment process, providing the possibility of therapeutic intervention to prevent establishment of persistent HPV infection.

## MATERIALS AND METHODS

### Cell culture.

Primary human foreskin keratinocytes (HFKs) were isolated with NIH Institutional Review Board approval and expanded in Rheinwald-Green F medium (3:1 ratio of Ham’s F-12 medium to high-glucose Dulbecco’s modified Eagle’s medium [DMEM], 5% fetal bovine serum [FBS], 0.4 µg/ml hydrocortisone, 8.4 ng/ml cholera toxin, 10 ng/ml epidermal growth factor, 24 µg/ml adenine, 6 µg/ml insulin, 1% glutamine) until passage 2 to 8 on a layer of lethally irradiated J2/3T3 murine fibroblasts. Antibiotics were not used. C33-A cervical carcinoma-derived cells were maintained in DMEM–10% FBS–1% glutamine–1% penicillin-streptomycin.

### Plasmids.

Wild-type HPV18 genomes, cloned into either pUC18 or pBR322, and an HPV18 mutant genome with a translational termination linker (TTL) at nucleotide position 2472 in the E1 gene have been described previously (42, 51, 52). Both single (R41A or I77A) and double (R41A/I77A) amino acid substitutions were generated in the E2 gene of HPV18 using the GeneArt mutagenesis kit, according to the manufacturer’s instructions (Invitrogen). The primer sets used for R41A were forward, 5′ TATTGGCAACTAATAGCTTGGGAAAATGCAAT 3′, and reverse, 5′ ATTGCATTTTCCCAAGCTATTAGTTGCCAATA 3′. The primer sets used for I77A were forward, 5′ AAAGCACATAAAGCTGCTGAACTGCAAATGGC 3′, and reverse, 5′ GCCATTTGCAGTTCAGCAGCTTTATGTGCTTT 3′.

Eukaryotic expression vector pMEP4-HPV18 E2 was generated by replacing the first 364 nucleotides of the E2 open reading frame with a codon-optimized fragment generated by gene synthesis. The E2 protein also encoded an N-terminal FLAG epitope tag. HPV18 E1-expressing pMEP9-E1 was generated by inserting recoded HPV18 E1 into the BamHI and HindIII site of the pMEP9 expression vector. Fragments containing E2 amino acid substitution R41A or I77A or double mutation R41A/I77A were also synthesized and substituted for the N-terminal region of E2 in pMEP4-HPV18 E2. *In vitro* transcription-translation vectors pTZ-Flag-E2 WT, R41A, and I77A and double mutation R41A/I77A were generated with a similar cloning strategy.

For reverse transcription-quantitative PCR (qRT-PCR) of viral mRNA, cDNA standards were synthesized for standard curves of HPV18 E1^E4 and E6*I, as described previously (42). pCMV-SPORT6-TBP was purchased from Open Biosystems (catalog no. MHS6278-202802567). The β-actin plasmid (eTC green fluorescent protein [GFP] beta-actin full length) used was purchased from AddGene (catalog no. 27123 [53]).

The pSheLL16 and 18 L1/L2 packaging plasmids were provided by Chris Buck (National Cancer Institute; http://home.ccr.cancer.gov/LCO).

### Recircularization of genomes.

Wild-type HPV18 genomes, cloned into either pUC18 or pBR322 (51, 52), were cleaved from their prokaryotic vectors with either NcoI or EcoRI, respectively, and recircularized by overnight ligation at 5 µg/ml DNA (54).

### RNA transfection.

Brd4 #6 and #9 siRNAs were purchased from Qiagen. HFKs were seeded at 25,000/well in a 24-well plate and transfected with 1 μl RNAiMax (diluted in 25 µl of Opti-MEM [Invitrogen]) into 0.5 ml of Rheinwald-Green F medium containing 20 nM siRNA, according to the manufacturer’s instructions (Invitrogen). Cell growth and morphology were monitored with a live-cell imaging system (IncuCyte; Essen BioScience, MI, USA). Images were collected every 4 h for 36 h.

### Quasivirus production.

HEK293TT cells were plated at 625,000/well in a 6-well dish. The next day, a single well was transfected with one of four different plasmid mixtures. All mixtures contained 0.8 µg of recircularized HPV18 genomes. In addition, the control plasmid mixture contained 0.8 µg each of empty vectors pMEP4 and pMEP9. The second mixture contained 0.8 µg pMEP4-E2, containing the HPV18 FLAG-tagged E2 gene and 0.8 µg empty pMEP9. The third mixture contained 0.8 µg pMEP9-E1 (harboring the HPV18 EE-tagged E1 gene), with 0.8 µg empty pMEP4. The last mixture contained 0.8 µg pMEP4-E2 and 0.8 µg pMEP9-E1. A 2.4-µg amount of plasmid mixtures was incubated with 5 µl Lipofectamine 2000 (Life Technologies) for 20 min in Opti-MEM (Life Technologies). The mixture was added dropwise to cells and allowed to incubate overnight. Total cellular DNA was isolated 48 h posttransfection with the DNeasy Blood and Tissue kit (Qiagen).

HPV18 quasiviruses were produced by cotransfecting codon-modified versions of both HPV18 and HPV16 L1 and L2 genes (pSHELL HPV16/18) with recircularized HPV18 genome. Forty-eight hours after transfection, cells were lysed in 0.5% Triton X-25 mM ammonium sulfate. Lysates were incubated at 37°C for 24 h and clarified by increasing the NaCl concentration to 850 mM. Viral particles were purified by ultracentrifugation through a 27 to 33 to 39% OptiPrep (Sigma-Aldrich) gradient. Fractions were collected, and an aliquot of each was analyzed by SDS-PAGE to identify those fractions that contain viral capsids (these were normally coincident with the 33 to 39% portion of the gradient) (Fig. 1A). Fractions that contained large amounts of L1 and L2 protein, along with cellular histones, were pooled into a working virus stock. To determine viral genome equivalents (VGE), HPV18 viral genomes were extracted from capsids in 100 µl digestion solution (20 mM Tris, pH 8, 20 mM dithiothreitol [DTT], 20 mM EDTA, 0.5% SDS, and 0.2% proteinase K) at 50°C for 20 min, purified using a QIAquick PCR purification kit (Qiagen), and quantified by qPCR as described below.

### Virus infections.

Cells were adsorbed for 1 h at 4°C with 50 to 100 VGE/cell of recombinant HPV18 quasivirus in 0.4 ml F medium/well of a 12-well dish. An 0.6-ml amount of F medium was added to each infection mixture, and cells were incubated until harvest.

### Neutralization assay.

Prior to infection, 100 VGE/cell quasivirus was incubated on ice in 0.4 ml of F medium with a 3-fold dilution series (1:150 to 1:328,050) of serum from rabbits pre- or post-immunization with Cervarix (gift from Patricia Day and John Schiller, National Cancer Institute). Each sample was used to infect one well of a 12-well dish, as described above.

### RNA extraction and qRT-PCR detection of viral transcripts.

Total RNA was isolated with the RNeasy mini-RNA extraction kit (Qiagen). RNA integrity was confirmed by capillary electrophoresis with RNA Nano Lab Chips on a Bioanalyzer (Agilent Technologies). Reverse transcriptase (RT) reactions were performed with the Transcriptor first-strand synthesis kit (Roche) with 1 µg total RNA, 60 µM random hexamers, and 2.5 µM oligo(dT) primers. Real-time qRT-PCR was performed with the ABI Prism 7900HT sequence detector (Applied Biosystems) and SYBR green PCR master mix (Roche). Each reaction mixture contained 1× SYBR green master mix, 1/20 of a cDNA preparation (synthesized from 1 µg of total RNA), and 0.3 µM (each) oligonucleotide primer in a total volume of 20 µl. PCR was performed in triplicate at 95°C for 15 min, followed by 40 cycles of denaturation at 95°C for 10 s and annealing and extension at 60°C for 30 s. In each run, a 10-fold dilution series (5 × 10^8^ to 5 × 10^1^ ag) of pUC57-E1^E4, pUC57-E6*I, or pDRIVE-TBP was included to generate a standard curve of cycle threshold versus log_10_ quantity (attograms). The specificity of each primer pair was determined by dissociation curve analysis. The sequences of the primers used are as follows: TBP fw, TAAACTTGACCTAAAGACCATTGCA; TBP rev, CAGCAAACCGCTTGGGATTA; HPV18 E6*I fw, CAAGACAGTATTGGAACTTACAGAGGTG; HPV18 E6*I rev, CTGGCCTCTATAGTGCCCAGC; HPV18 E1^E4 fw, CAACAATGGCTGATCCAGAAGTAC; HPV18 E1^E4 rev, TAGGTCTTTGCGGTGCCC; BRD4 fw, ACCAGTTTGCATGGCCTTTC; BRD4 rev, AATGATCTTATAGTAATCAGGGAGGTTCA.

### HPV18 genome copy number analysis.

Total cellular DNA was isolated from keratinocytes with the DNeasy Blood and Tissue kit (Qiagen). Copy number analysis was conducted by comparing the unknown samples to standard curves of HPV18 DNA. The β-actin DNA copy number was used as an endogenous control. The sequences of the primers used were described previously (42).

### Replication assay.

DNA was digested prior to qPCR or Southern blot analysis. An 0.5-µg amount of total DNA was digested with EcoRI, which cuts the viral genome once, and one of the following: DpnI (to digest unreplicated bacterial DNA) or MboI (to digest DNA replicated in eukaryotes).

### Southern blot analysis.

After digestion, samples were separated in an 0.8% Tris-acetate-EDTA (TAE) agarose gel. DNA was visualized with 0.5 µg/ml ethidium bromide and transferred onto nylon membranes with a TurboBlotter downward transfer system (Whatman). Membranes were UV cross-linked, dried, incubated with prehybridization buffer, and incubated overnight with [^32^P]dCTP-labeled HPV18 DNA (generated from 25 ng of twice-gel-purified linear HPV18 DNA with the Random Prime DNA labeling kit [Roche]) in hybridization buffer (0.75× SSC [1× SSC is 0.15 M NaCl plus 0.015 M sodium citrate], 2% SDS, 5× Denhardt’s solution, 0.2 mg/ml sonicated salmon sperm DNA). Membranes were washed with 0.1% SDS-0.1× SSC. Hybridized DNA was visualized and quantitated by phosphorimaging on a Typhoon scanner (GE Bioscience).

### SDS-PAGE and Western blotting.

Cells were lysed in SDS extraction buffer (50 mM Tris-HCl, pH 6.8, 5% SDS, 10% glycerol). Protein concentration was determined with a bicinchoninic acid (BCA) protein assay kit (Thermo-Pierce), samples were incubated in 20 µM DTT and 1× lithium dodecyl sulfate (LDS) buffer (Invitrogen) at 72°C for 10 min, and 10 µg total protein was separated on a 4 to 12% bis-Tris polyacrylamide gel (Invitrogen). Proteins were transferred overnight onto a polyvinylidene difluoride (PVDF) membrane (Millipore) and subsequently immunoblotted. The antibodies used mouse antitubulin (Sigma catalog no. T5168; 1:10,000) and FLAG-M2 (Sigma catalog no. F1802; 1:5,000). The C-terminal region-specific Brd4 antiserum MCB2 was raised in rabbits against a C-terminal peptide of Brd4 and affinity purified (MCB2; 1:2,000) (40). Species-appropriate secondary antibodies conjugated to horseradish peroxidase (Pierce) were used at a 1:10,000 dilution and detected with SuperDura Western detection reagent (Thermo-Pierce).

### E2 protein expression levels.

To generate cell lines expressing inducible WT and mutated E2 proteins, 5 × 10^5^ C33-A cells were seeded onto a 10-cm dish in antibiotic-free medium and cultured for 24 h. The next day, 18 µl of Fugene-6 transfection reagent (Promega) and 6 µg of pMEP4-HPV18 E2 WT (or single/double site mutant) plasmid DNA was added to 0.6 ml of optimum serum-free medium (Invitrogen), mixed, incubated at room temperature for 30 min, and added dropwise to each plate of cells. The following day, cells were trypsinized and transferred to a T175 flask in medium containing 80 µg/ml hygromycin B and 1% penicillin-streptomycin. Hygromycin selection was maintained for 2 weeks with fresh medium changes every 3 to 4 days, after which colonies were pooled and expanded. To measure E2 protein levels, 4 × 10^6^ cells from each line were seeded onto a 10-cm dish. The next day, cells were induced to express E2 by the addition of 3 µM cadmium sulfate for 4 h. Cells were rinsed with ice-cold phosphate-buffered saline (PBS) and scraped into 1 ml NP-40 lysis buffer (50 mM HEPES, pH 7.4, 100 mM NaCl, 1.5 mM MgCl_2_, 2 mM EDTA, 0.25% NP-40, 1× Roche protease inhibitor cocktail). Cells were gently lysed by rocking at 4°C for 30 min. Lysates were clarified by centrifugation at 12,000 × *g* for 10 min. Samples were prepared for Western blot analysis by mixing 100 µl lysate with an equal volume of SDS extraction buffer (50 mM Tris-HCl, pH 6.8, 5% SDS, 10% glycerol). Samples were incubated at 100°C for 3 min with 1× LDS sample buffer (Invitrogen) containing 20 µM DTT. Twenty-microliter samples were resolved by SDS-PAGE, and Western blot analysis was conducted as described above.

### E2-Brd4 binding assay.

[^35^S]methionine-labeled wild-type and mutated E2 proteins were *in vitro* translated from pTZE2 plasmids using the TNT T7 quick-coupled reticulate lysate system (Promega). The concentration of each E2 protein was quantitated using a Typhoon phosphorimager and was normalized using labeled no-E2 reaction mixtures. FLAG-tagged Brd4 was expressed and purified from SFf9 insect cells, as described elsewhere (15). Five microliters of Brd4 was mixed with 5 µg of purified rabbit IgG (Jackson Laboratories) or 3 µl 2290 Brd4 antiserum (40, 55), a gift from Keiko Ozato, National Institute of Child Health and Human Development, in 100 µl of PBS–10% glycerol–0.1% Triton X-100 and incubated overnight at 4°C. A 12.5-µl amount of ^35^S-E2 protein was added to the Brd4 or control immune complexes and incubated for 1 h at room temperature with mixing. E2-Brd4 complexes were then isolated with 25 µl preequilibrated Dynabeads protein G (Invitrogen) for 1 h at room temperature with mixing. Proteins were eluted in protein sample buffer (1× LDS buffer, 20 µM DTT [Invitrogen]), incubated at 100°C for 3 min, and separated by SDS-PAGE on a 4 to 12% bis-Tris polyacrylamide gel (Invitrogen). Gels were fixed in 10% acetic acid-40% methanol for 1 h, enhanced in Amplify solution (GE Healthcare) for 20 min, reinforced in 10% glycerol for 5 min, dried, exposed to a Typhoon phosphorimager, and quantified with ImageQuant TL.

## SUPPLEMENTAL MATERIAL

Figure S1  Effect of Brd4 downregulation on cell growth. HFKs were transfected with 20 nM Brd4-targeting or All* negative-control siRNA. Twenty-four hours post-transfection, cells were infected with 100 VGE/cell of HPV18 quasivirus. Cell growth was monitored in an IncuCyte microscope to assess toxicity of the Brd4 downregulation. Proliferation was measured (percent confluence) in an IncuCyte microscope for up to 72 h (note that keratinocytes are cultured on a background of irradiated feeders). The data shown are compiled from four technical replicates (individual wells) and are from a representative experiment from two independent experiments. Error bars show standard errors of the means. Download Figure S1, PDF file, 0.2 MB

Figure S2  Brd4 depletion does not alter infection by quasiviruses as determined by amount of viral DNA delivered to the cell. HFKs were transfected with 20 nM Brd4-targeting or All* negative control siRNA. Twenty-four hours post-transfection, cells were infected with 100 VGE/cell of HPV18 quasivirus. Seventy-two hours post-infection, cells were harvested for DNA. Total DNA was either undigested (total DNA) or digested with DpnI (replicated DNA). The abundance of viral DNA was measured by qPCR and normalized with β-actin levels. Unreplicated DNA was calculated as total DNA − replicated DNA. All values were normalized to siCtrl total DNA. *n* = 3. Error bars show standard errors of the means. These data are an expanded version of the data shown in Fig. 4F. A *t* test showed no statistical difference (ns) between the unreplicated DNA samples. Download Figure S2, PDF file, 0.2 MB

Movie S1  Effect of Brd4 downregulation on cell growth. HFKs were transfected with 20 nM Brd4-targeting or All* negative-control siRNA. Twenty-four hours post-transfection, cells were infected with 100 VGE/cell HPV18 quasivirus (time = 0 h). Cell growth was monitored in an IncuCyte microscope to assess toxicity of the Brd4 downregulation. Phase-contrast images were taken every 4 h until time of harvest (*t* = 72 h) and compiled into a movie. Note that keratinocytes are cultured on a background of irradiated feeders. Download Movie S1, AVI file, 13.6 MB
