# Supplementary material for: Brd4 Activates Early Viral Transcription upon Human Papillomavirus 18 Infection of Primary Keratinocytes
Source: mBio. 2016 Nov 22;7(6):e01644-16. doi: 10.1128/mBio.01644-16 (PMC5120138; doi:10.1128/mBio.01644-16)
Supplement: Figure S1 — Effect of Brd4 downregulation on cell growth. HFKs were transfected with 20 nM Brd4-targeting or All* negative-control siRNA. Twenty-four hours post-transfection, cells were infected with 100 VGE/cell of HPV18 quasivirus. Cell growth was monitored in an IncuCyte microscope to assess toxicity of the Brd4 downregulation. Proliferation was measured (percent confluence) in an IncuCyte microscope for up to 72 h (note that keratinocytes are cultured on a background of irradiated feeders). The data shown are compiled from four technical replicates (individual wells) and are from a representative experiment from two independent experiments. Error bars show standard errors of the means. Download [file mbo006163082sf1.pdf]

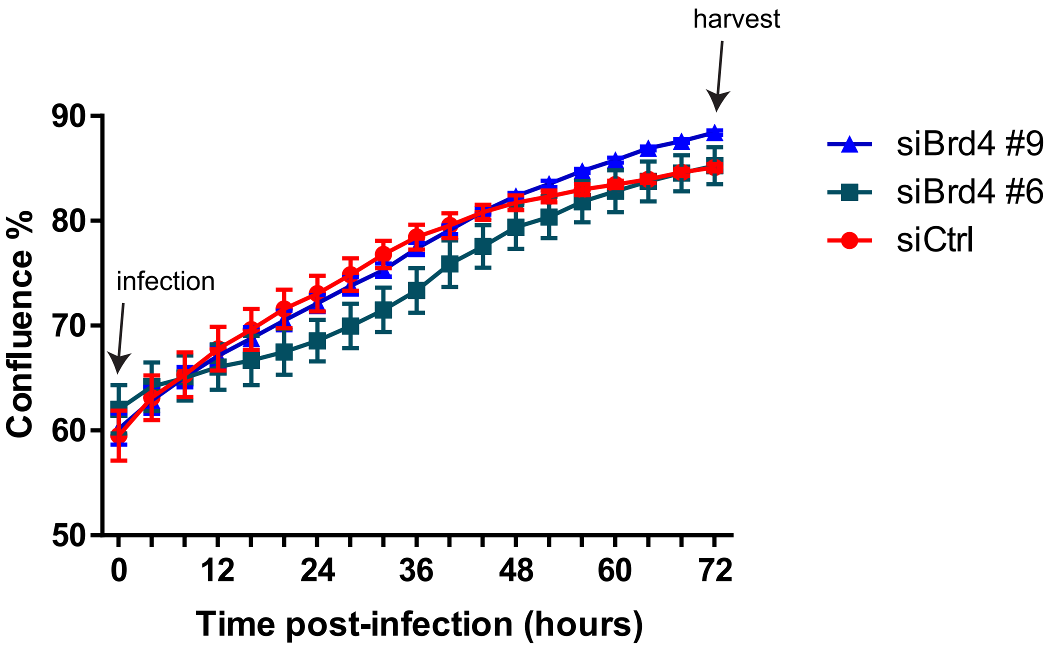

Supplementary Figure 1 Effect of Brd4 downregulation on cell growth

HFKs were transfected with 20nM Brd4-targeting or All* negative control siRNA. Twenty-four hours post-transfection, cells were infected with 100VGE/cell HPV18 quasivirus. Cell growth was monitored in an Incucyte microscope to assess toxicity of the Brd4 downregulation. Proliferation was measured (% confluence) in an Incucyte microscope for up to 72 hours (note that keratinocytes are cultured on a background of irradiated feeders). The data shown is compiled from four technical replicates (individual wells) and is a representative experiment from two independent experiments. Error bars=SEM
