# Supplementary material for: Brd4 Activates Early Viral Transcription upon Human Papillomavirus 18 Infection of Primary Keratinocytes
Source: mBio. 2016 Nov 22;7(6):e01644-16. doi: 10.1128/mBio.01644-16 (PMC5120138; doi:10.1128/mBio.01644-16)
Supplement: Figure S2 — Brd4 depletion does not alter infection by quasiviruses as determined by amount of viral DNA delivered to the cell. HFKs were transfected with 20 nM Brd4-targeting or All* negative control siRNA. Twenty-four hours post-transfection, cells were infected with 100 VGE/cell of HPV18 quasivirus. Seventy-two hours post-infection, cells were harvested for DNA. Total DNA was either undigested (total DNA) or digested with DpnI (replicated DNA). The abundance of viral DNA was measured by qPCR and normalized with β-actin levels. Unreplicated DNA was calculated as total DNA − replicated DNA. All values were normalized to siCtrl total DNA. n = 3. Error bars show standard errors of the means. These data are an expanded version of the data shown in Fig. 4F. A t test showed no statistical difference (ns) between the unreplicated DNA samples. Download [file mbo006163082sf2.pdf]

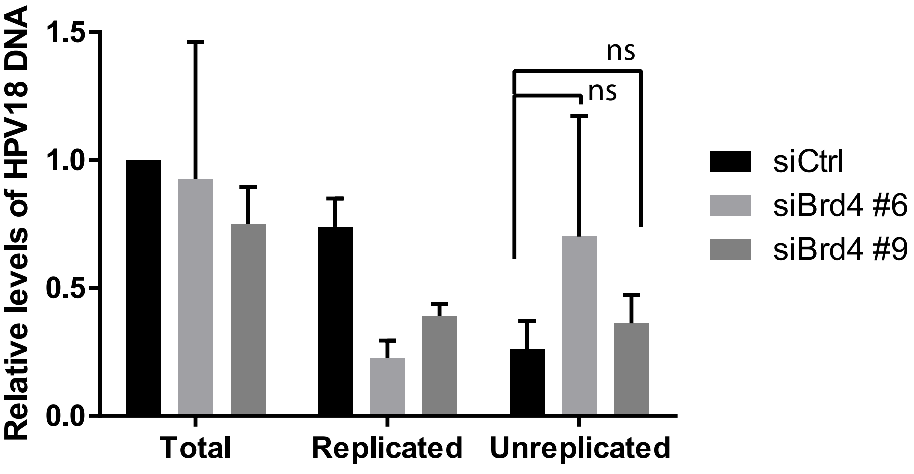

Supplementary Figure 2
Brd4 depletion does not alter infection by quasiviruses as determined by amount of viral DNA delivered to the cell

HFKs were transfected with 20nM Brd4-targeting or All* negative control siRNA. Twenty-four hours post-transfection, cells were infected with 100VGE/cell of HPV18 quasivirus. 72 hours post infection, cells were harvested for DNA. Total DNA was either undigested (Total DNA) or digested with DpnI (Replicated DNA). The abundance of viral DNA was measured by qPCR and normalized with β-actin levels. Un-replicated DNA was calculated as Total DNA – Replicated DNA. All values were normalized to siCtrl Total DNA. N=3. Error bars= SEM. This data is an expanded version of the data shown in Figure 4F. A t-test showed no statistical difference (ns) between the un-replicated DNA samples.
